# Role of Energy Metabolism in the Progression of Neuroblastoma

**DOI:** 10.3390/ijms222111421

**Published:** 2021-10-22

**Authors:** Monika Sakowicz-Burkiewicz, Tadeusz Pawełczyk, Marlena Zyśk

**Affiliations:** Department of Molecular Medicine, Medical University of Gdansk, 80-211 Gdansk, Poland; monika.sakowicz-burkiewicz@gumed.edu.pl (M.S.-B.); tadeusz.pawelczyk@gumed.edu.pl (T.P.)

**Keywords:** neuroblastoma, glycolysis, extracellular vesicles

## Abstract

Neuroblastoma is a common childhood cancer possessing a significant risk of death. This solid tumor manifests variable clinical behaviors ranging from spontaneous regression to widespread metastatic disease. The lack of promising treatments calls for new research approaches which can enhance the understanding of the molecular background of neuroblastoma. The high proliferation of malignant neuroblastoma cells requires efficient energy metabolism. Thus, we focus our attention on energy pathways and their role in neuroblastoma tumorigenesis. Recent studies suggest that neuroblastoma-driven extracellular vesicles stimulate tumorigenesis inside the recipient cells. Furthermore, proteomic studies have demonstrated extracellular vesicles (EVs) to cargo metabolic enzymes needed to build up a fully operative energy metabolism network. The majority of EV-derived enzymes comes from glycolysis, while other metabolic enzymes have a fatty acid β-oxidation and tricarboxylic acid cycle origin. The previously mentioned glycolysis has been shown to play a primary role in neuroblastoma energy metabolism. Therefore, another way to modify the energy metabolism in neuroblastoma is linked with genetic alterations resulting in the decreased activity of some tricarboxylic acid cycle enzymes and enhanced glycolysis. This metabolic shift enables malignant cells to cope with increasing metabolic stress, nutrition breakdown and an upregulated proliferation ratio.

## 1. Neuroblastoma, a Pediatric Heterogeneous Solid Tumor—Overview

Neuroblastoma is a solid childhood cancer, causing up to 15% of childhood cancer-related deaths [1]. Despite multidisciplinary approaches, still no effective treatment strategy has been suggested for clinical applications. Neuroblastoma is a solid tumor, which manifests variable clinical behaviors ranging from spontaneous regression to widespread metastatic disease [2]. Neuroblastoma arises from neural crest stem cells and matures into various kinds of nerve tissues, e.g., cranial neurons and glia, bone cells or peripheral sympathetic neurons and Schwann cells [3]. Cohort studies have proven that a significant number of high-risk cases have genetic alterations, e.g., the amplification of *MYCN*, mutations of the *ALK* gene or germline mutations in *SDHA* and *SDHB* genes [4]. Mutations in *SDHA* and *SDHB* genes suppress succinate dehydrogenase activity and promote the metabolic shift from complex and efficient mitochondrial energy production pathways to cytoplasmic glycolysis [1,2,5,6]. This phenomenon, known as the Warburg effect, plays a pivotal role in the growth of the solid tumor [1,2,5,6]. Due to the crucial role of energy metabolism in neuroblastoma tumorigenesis, the overarching purpose of this review is to provide a comprehensive update of the current state of knowledge on the role of energy metabolism on neuroblastoma tumorigenesis, pointing to their potential effect on the spreading of neuroblastoma malignancy.

## 2. Neuroblastoma and Extracellular Vesicles

In recent years, in vitro studies have indicate that, similar to other normal cells and cancer cells, neuroblastoma (NB) release extracellular vesicles (EVs) to communicate with the microenvironment [6]. Marimpietri and colleagues isolated extracellular vesicles in a size of less than 80 nm from neuroblastoma cell lines: SH-SY5Y, IMR-32, GI-LI-N and HTLA-230 7. They found NB-derived extracellular vesicles packed with proteins known to modulate the tumor microenvironment and promote tumor progression [7]. The same studies found a wide range of metabolic enzymes and other proteins suggested as a potential NB fingerprint [7]. Two years later, Challagundla and collaborators showed NB-derived EVs to contain miR21, an RNA molecule supporting the chemotherapy resistance of the tumor [8]. Finally, earlier this year, Esposti and collaborators suggested exosomal DNA as an alternative marker for NB molecular diagnostics, which might in the future become a substitute for liquid biopsy [9]. This growing body of evidence concerning exosomes involved in NB tumorigenesis has attracted researchers’ attention and resulted in a body of data linking exosomes, tumorigenesis and energy metabolism.

## 3. Extracellular Vesicles: Definitions and Subtypes

Tumor-derived extracellular vesicles are often called cell–cell crosstalk bridges, which facilitate the communication of donor cancer cells with healthy recipients [10,11]. EVs can contact neighbor cells as well as pass through the blood stream and reach distant recipients [12]. This general definition includes a heterogenous group of cellular vesicles, which have been classified further by vesicle size and function [13].

Apoptotic bodies are the largest extracellular vesicles produced within apoptosis [13]. Apoptotic bodies are between 1 and 5 µm; thus, they can be identified under light microscopy [13]. Apart from small molecules such as proteins or nucleic acids, apoptotic extracellular bodies (Apo-EVs) have been proven to carry larger cargoes, such as mitochondria, nuclear fragments or endoplasmic reticulum [14]. So far, little is known about the role of apoptotic bodies in tumorigenesis, although some studies hypothesize that dyeing tumor cells, affected by radio- or chemotherapy, can spread apoptotic EVs in order to repopulate malignant cells [15]. A mechanism of apoptosis-dependent cancer cell growth stimulation has been proposed by Huang and colleagues, who co-cultured irradiated cells with 4T1 breast cancer cells and noted an increased 4T1 cell proliferation [16]. The role of apoptotic bodies in this stimulation was proven by Pavlyukov and colleagues, who conditioned glioblastoma cells with apoptotic bodies isolated from irradiated glioblastomas [15]. Several studies showed SH-SY5Y cells (in an in vitro model of human neuroblastoma) to produce apoptotic bodies in response to cytotoxic conditions [17,18]. However, the role of apoptotic bodies in the progression of neuroblastoma has not been determined yet.

Oncosomes are defined as EVs which are heterogenous in size but consistent in function [19]. Small oncosomes are classified as microvesicles ranging between 100 and 400 nm, while the diameter of large oncosomes ranges between 1 and 10 µm [19]. Oncosomes have been proven to cargo oncoproteins (e.g., EGFRvIII in gliomas [20]) and oncogenes (e.g., *MYC* in prostate cancer [21]). All oncosomes have one fundamental feature—they originate from the plasma membrane of cancer cells [19]. To the best of our knowledge, NB-derived extracellular vesicles have not been named as *oncosomes*. However, proteomic and genomic data have confirmed that these EVs are packed with oncogenes; thus, they meet the definition of *oncosomes* [22]. It has been shown that exosomes isolated from *MYCN*-amplified SH-SY5Y cells cargo miRNAs linked with AHR signaling, supporting aggressive tumorigenesis [22].

Microvesicles are significantly smaller than apoptotic bodies (1–0.05 µm) and are shed from the outward budding of the plasma membrane [12], while exosomes are the smallest of the currently known extracellular vesicles [12]. Exosomes originate from multivesicular bodies and are formed during complex processes starting inside the cells [23]. EVs ranging between 30 and 150 nm in size are defined as exosomes [12]. Fixed exosomes, visualized by electron microscopy, have a characteristic cup-like shape. The overlap between smaller microvesicles and exosomes limits isolation techniques [12]. Ultracentrifugation is the most common exosome isolation technique, but the obtained fractions only distinguish larger microvesicles from a mixture of exosomes and smaller microvesicles. The limited exosome isolation results in studies showing the molecule content profile or biological functions of mixed extracellular vesicles, including both exosomes and smaller microvesicles [24]. Exosomes are widely understood as pivotal players in tumorigenesis, although in the majority of reports, exosomes come from ultracentrifugation [12]. Thus, in this review, they were called the EV fraction instead of pure exosomes.

Exosome production starts from endocytic vesicles transporting their cargoes to the main sorting platforms, known as early endosomes [12,25,26]. From this point, maturating endosomes recover material useful in further cellular processes and dispatch their remaining cargo to secretion pathways [12,25,26]. Late endosomes have an acidic core filled with vesicles [12,25,26]. Finally, the matured endosomes, called multivesicular bodies, can fuse with the cellular plasma membrane to release exosomes [12,25,26].

It has been suggested that donor cancer cells may adapt the exosomal cargo to the recipient cells, although the exact mechanism remains unknown [12,27,28]. For instance, the exosomes isolated from gastric and pancreatic cancers are involved in autocrine pathways stimulating the abnormal proliferation of neighbor cancer cells [29,30]. The overall exosomal cargoes include lipids, proteins, mRNA and miRNA [10,25]. Exosomal membranes are rich in cholesterol, sphingolipids, glycerophospholipids and ceramide [31,32]. Proteins are localized either in the exosomes core or inside the exosomal membranes. Proteins are categorized by their functions: endosomal markers (tetraspanins, e.g., CD9, CD63, CD81, CD82), membrane transport and fusion proteins (Rabs, flotillin, annexins), multivesicular body biogenesis proteins (e.g., Alix, TSG101), cytoskeleton proteins (actin, tubulin), growth factors, heat-shock proteins (e.g., HSP90, HSC60), adhesion proteins (e.g., integrins, ICAM1), metabolic enzymes (pyruvate dehydrogenase complex, tricarboxylic acid enzymes, electron transport chain enzymes, glycolysis enzymes, fatty acids β-oxidation enzymes, malate-aspartate enzymes, etc.) and others [32,33,34].

### 3.1. Extracellular Vesicles Contribute to Neuroblastoma Tumorigenesis and Energy Metabolism

EVs are hypothesized to mediate a wide array of pathological scenarios, such as the suppression of the immune response, development of drug resistance, stimulation of angiogenesis and tumorigenesis [27,31,35]. A growing body of evidence shows that NB-driven EVs contain oncogenic miRNAs, which can contribute to tumorigenesis [22]. Exosomes, isolated from NB patients’ plasma, contain has-miR199a-3p, a miRNA regulating *NEDD4* expression, in order to promote tumor proliferation and migration [36]. Exosomal mi-155 has been identified in NB patients and shown in vitro to participate in chemotherapy resistance signaling [8]. As Colletti and collaborators showed, exosomal miR375 is involved in the NB infiltration of bone marrow [11]. Several other known oncogenic miRNAs have been profiled in exosomes secreted by SK-N-E(2)-C and Kelly neuroblastoma cell lines [22].

**Figure 1:**
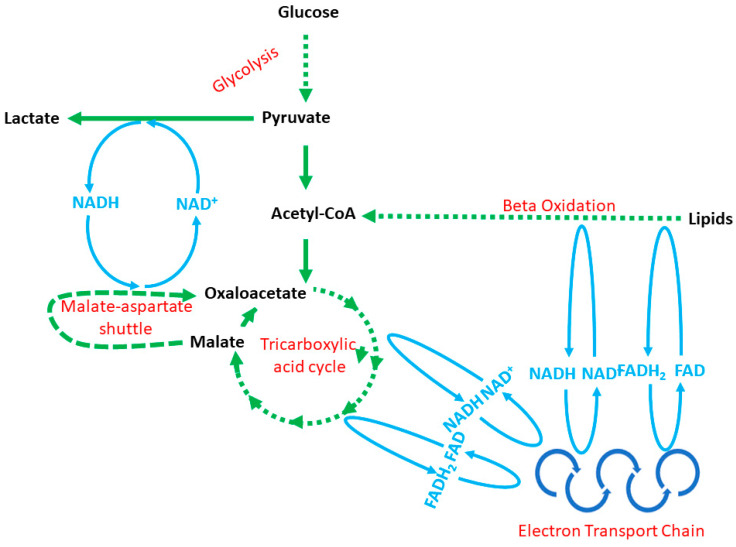
The relationships between energy production pathways: cytoplasmic glycolysis, the mitochondrial tricarboxylic acid cycle, the mitochondrial electron transport chain, the malate–aspartate shuttle and mitochondrial and/or peroxisomal fatty acid β-oxidation. Note: the NADH/NAD^+^ and FADH_2_/FAD blue reaction lines do not reflect the exact place in a particular cycle, but show the connections existing between metabolic pathways.

Tumorigenesis is a high-energy-consuming process requiring NB cells to adapt energy metabolism to dynamic changes in tumor mass [37]. Recent reports show that, apart from oncogenic nucleic acids, NB-derived exosomes harbor a significant number of metabolic enzymes involved in glycolysis, the tricarboxylic acid cycle, the electron transport chain, fatty acids β-oxidation, malate–aspartate shuttle, glutaminolysis and others (Figure 1, Table 1) [6,7,38]. These enzymes can easily build up fully operative metabolic network capabilities (Figure 1). These enzymes have been profiled with a proteomic assessment, although to the best of our knowledge, the metabolic activity of EV-derived enzymes has not been considered in published reports yet. Thus, for the time being, we can only speculate about EV involvement in the tumor-dependent modification of metabolic networks in the tumor microenvironment. Furthermore, at the time of this writing, there were five reports in which NB-derived EVs were analyzed with proteomics, and a few dozen projects looking for the answer to the question of what role EVs play in tumorigenesis [6,7,35,39,40]. Furthermore, it seems that the content of EVs, tumorigenesis and energy metabolism is tightly linked with one another, which we summarized further in this Section.

### 3.2. Lipolysis and Fatty Acid β-Oxidation

Several cancer cells (e.g., breast, prostate and colon cancers) have been proven to grow in the neighborhood of adipocytes [41]. Adipocytes are fat cells which manage energy storage and utilization by balancing between lipogenesis and lipolysis pathways. In clinical practice, cancer patients often suffer from adipocyte atrophy, which comes from an overactive lipolysis and energy release [41]. Released fatty acids can be incorporated by neighbor cells to conduct energetically favorable β-oxidation [41]. Pancreatic cancer cells have been proven to secrete EVs rich in adrenomedullin, a peptide-mediating lipolysis, while breast cancer cells reprogram adipocyte metabolism to a catabolism pathway transferring exosomal miRNA-126 [42,43].

For years, glucose oxidation attracted the most attention in tumorigenesis studies, while fatty acid β-oxidation was a minor point [44]. Furthermore, in the sphere of lipid metabolism, rather than fatty acid β-oxidation, de novo fatty acid production was believed to play an important role [44]. However, recent studies underline the crucial role of fatty acid β-oxidation, which provides NADH and FADH2 molecules for oxidative phosphorylation (OXPHOS). Other benefits of fatty acid metabolism are: (1) high efficiency in energy production; (2) metabolism conducted in mitochondria as well as in peroxisomes [44]. Peroxisomes are significantly smaller than mitochondria; therefore, they can easily re-localize to the place where they are needed most. A proteomic analysis of neuroblastoma-derived EVs has confirmed the presence of acidic calcium-independent phospholipase A2 and phospholipase C, enzymes involved in lipolysis and free fatty acid release from adipocytes [6,7]. Furthermore, NB-derived EVs support not only lipolysis, but also lipid uptake (long-chain fatty acid transport protein 3 and 4) and transport (albumin and fatty acid-binding proteins) [6,45]. Once the cell uptakes fatty acids, these must be stored in lipid droplets to avoid lipotoxicity [6,45]. Here, EV-derived proteins preliminarily convert fatty acids to diacylglycerols and triacylglycerols (acyltransferases) and further organize them into lipid droplets (perilipin 2 and 3, caveolin-1) [6,45]. To support such a fatty acid restoration, NB donor cells send lipases and protein kinase A [6,7]. Finally, the presence of fatty acid β-oxidation enzymes working in both peroxisomes and mitochondria has been widely identified in neuroblastoma cell-driven exosomes [6,7,35].

### 3.3. Glycolysis

It is worth mentioning that phospholipase C was identified in exosomes shed by N-Myc-amplified SK-N-BE2 neuroblastoma cells [7]. *MYCN* gene amplification correlates with a poor diagnosis for NB patients and the upregulation of enzymes involved in fatty acid β-oxidation [7,46]. The same genetic aberration (*MYCN* amplification) is responsible for the Warburg effect oxidation, in which malignant cells shift energy metabolism to glycolysis [7,46].

The main goal of energy metabolism is to generate a molecular fuel supplying the basic cellular processes [47]. Under glycolysis, healthy cells utilize glucose to generate pyruvate, which can further enter the mitochondrial tricarboxylic acid cycle linked with oxidative phosphorylation (OXPHOS) or stay in the cytoplasm to be converted to lactate (anaerobic reaction) [47]. In general, healthy cells prefer OXPHOS over glycolysis, although the exact preferences differ and depend on the cell functions [47,48]. Dissimilar to healthy cells looking for an effective energy metabolism, cancer cells are more “wasteful”. Malignant cells have to fill energy needs resulting from an elevated proliferation, migration and other tumor cell activities linked with tumorigenesis. Thus, tumor cells shift energy metabolism from the complex tricarboxylic acid cycle with OXPHOS to the less demanding glycolysis [45]. Interestingly, such a metabolic shift is not moderated by oxygen availability, as initially believed [49,50]. There are two working theories trying to explain such phenomena—the classical Warburg theory and cancer cell symbiosis [49,50]. The first theory perfectly covers the tumorigenesis which takes place under hypoxic conditions. A rapid tumor growth leads to glucose and oxygen shortages in the tumor core [49,50]. To keep up with energy needs, cancer cells shift energy metabolism to anaerobic lactate production [49,50]. Recent reports show that cancer cells can develop more complex symbiosis pathways between cancer cells. In detail, donors produce energy and lactate under glycolysis, while the recipients turn lactate into pyruvate and proceed with the tricarboxylic acid cycle [47,49,50]. Nevertheless, anaerobic metabolism is the main player in tumorigenesis, as reflected in proteomic studies. All data coming from such an analysis of NB-driven exosomes confirmed have the presence of glycolytic enzymes [6,7,35,39]. Furthermore, Keerthikumar and colleagues identified 39 proteins packed in exosomes shed from SH-SY5Y, which are directly involved in glycolysis [40]. NB is not alone in favoring the glycolysis pathway. The previously mentioned exosomal miR-155 (identified in *MYCN*-amplified SK-N-BE(2)) is also secreted by melanoma to promote glycolysis in stromal fibroblasts [51]. About 5% of exosomal proteins secreted by other *MYCN*-amplified B7-H3 medulloblastoma (an aggressive pediatric cancer localized in the central nervous system) has been associated with glycolysis [52]. Extracellular-vesicle-encapsulated miR-105 originating from breast cancer cells activates MYC signaling and induces glycolysis [53]. In other words, EVs cargo a wide range of molecules (proteins and nucleic acids) to shift the energy metabolism to glycolysis.

### 3.4. Tricarboxylic Acid Cycle and Oxidative Phosphorylation

Pyruvate produced in glycolysis must be oxidized by the pyruvate dehydrogenase complex to acetyl-CoA (Figure 1) [47,48]. As reported above, cancer cells prefer glycolysis and β-oxidation rather than the tricarboxylic acid cycle and OXPHOS. Indeed, glycolytic enzymes are the most frequently identified exosomal proteins in the ExoCarta database [54]. Studies on tumorigenesis, in general, agree that malignant cells promote a metabolic shift to glycolysis. The role of exosomes in the tricarboxylic acid cycle and OXPHOS remains largely unknown. Oliynyk and colleagues showed that in SK-N-BE(2) and Kelly cells, the amplification of the *MYCN* oncogene enhanced both glycolysis and OXPHOS activity [55]. Proteomic studies showed NB-derived exosomes to harbor metabolic enzymes involved in the tricarboxylic acid cycle and OXPHOS [6,7,35,39]. However, the same studies point out the considerably lower occurrence of these enzyme compared to glycolytic proteins [6,7,35,39]. Furthermore, Diehl and colleagues proved that in the progression of colorectal cancer, a cancer-associated fibroblast promotes malignancy through the stimulation of the tricarboxylic acid cycle with OXPHOS [56].

## 4. The Neuroblastoma and MYC Family

The amplification of the *MYCN* oncogene correlates with a poor diagnosis in onco-pediatric NB patients [1,2,35,39]. Encoded by *MYCN*, the N-MYC protein controls cell growth and energy metabolism [37,48,57,58]. As mentioned, N-MYC dysregulates fatty acid β-oxidation and glycolysis in neuroblastoma patients. However, the involvement of the *MYCN* oncogene is not limited only to these metabolic pathways. A growing body of evidence indicates that *MYCN* amplification helps NB to cope with energy needs via glutamine metabolism [59,60]. *MYCN*-amplified Tet21N and SK-N-BE(2) neuroblastomas have been proven to develop two alternatives [56,61]. They stimulate either the glutamine uptake or de novo synthesis [56,61]. The overexpression of *MYCN* upregulates the metabolic genes promoting glutaminolysis, which converts glutamine into glutamate [60,62]. Next, glutamine metabolism starts from glutaminolysis glutamate [60,62]. To enter the tricarboxylic acid cycle, glutamate is converted by aspartate aminotransferase into α-ketoglutarate, an enzyme widely spread by *MYCN*-amplified NB [6,39,60]. In NB, genetic aberrations include mutations in the *IDH1* and *2* genes, which leads to the α-ketoglutarate-triggered accumulation of an oncometabolite called D-2-hydroglutarate, described in the section below.

## 5. Mutations in Tricarboxylic Acid Cycle Enzymes Promote Glycolysis in Neuroblastoma Cells

**Figure 2:**
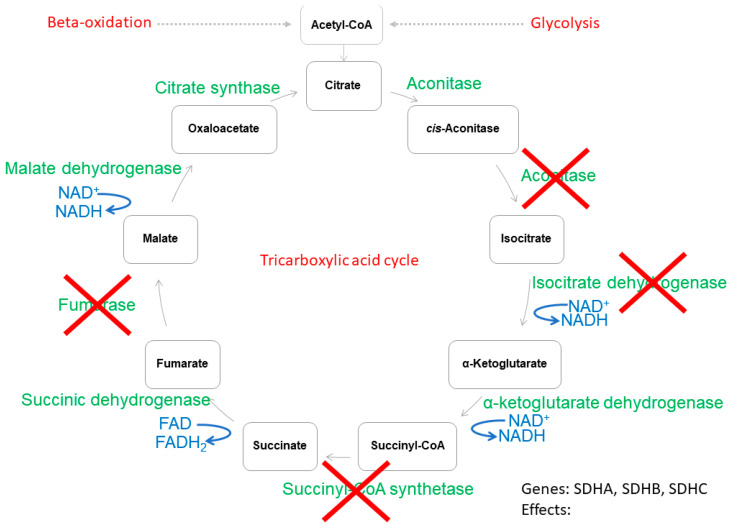
The tricarboxylic acid cycle and common enzyme mutations. Colors: red crosses show inactive enzymes (green), nucleotides (blue), metabolites (black). Each red-crossed enzyme has a comment with the name of the gene being mutated and the metabolic outcomes rising from such mutation. Please note that this figure reports only alterations in genes encoding mitochondrial enzyme isoforms.

As shown above, energy metabolism plays a crucial role in the progression of tumorigenesis. Thus, in this section we focused on cancer cells and discussed the most common mutations in genes encoding tricarboxylic acid cycle enzymes (Figure 2). We stayed with the general hypothesis that malignant cells promote a metabolic shift (from mitochondrial aerobic processes to glycolysis) to initiate a hypoxia-like cell response.

Aconitase is a tricarboxylic acid enzyme, converting citrate to isocitrate (Figure 2) [38,47,63]. This enzyme is known to have two isoforms, mitochondrial (encoded by the *ACO2* gene) and cytoplasmic (encoded by the *ACO1* gene) [38,47,64]. Alterations in *ACO1* have been identified in breast cancer, although no significant impact of this mutation has been noted [65]. Conversely, mutations in the *ACO2* gene result in serious cellular outcomes. For example, prostate cancer cells carrying the *ACO2* mutation have a significantly less active aconitase, which limits citrate/isocitrate conversion. Cancer cells with such a genetic alteration are known to secrete a great amount of citrate [66]. Other processes leading to poor aconitase activity and citrate accumulation have also been noted in prostate cancer [67]. In this case, no alteration in the *ACO2* gene was reported, but p53 overactivation downregulated the *ACO2* gene expression [67]. Accumulated citrate can be utilized to restore cytosolic acetyl-CoA, which, additionally, can be introduced to lipogenesis (Figure 2) [63,64].

The isocitrate dehydrogenase enzyme converts isocitrate to α-ketoglutarate in the cytoplasm (gene *IDH1*) or mitochondria (genes: *IDH2*, *IDH3*) (Figure 2). Mutations in the *IDH1* and *IDH2* genes are commonly diagnosed in oncological patients with glioblastomas [68]. Mutated isocitrate dehydrogenase displays less activity against isocitrate [48,69,70]. Isocitrate is a catalytic homodimer; in the case of mutation, it can either remain a homodimer (having two mutated monomers exhibiting no catalytic activity) or become a heterodimer with one wild-type and one mutated monomer [48,69,70]. Studies with primary and secondary gliomas have shown that tumors have heterodimers which exhibit neomorphic activity [48,69,70]. Mutants, instead of catalyzing the isocitrate conversion, consume α-ketoglutarate to produce an onco-metabolite called D-2-hydroglutarate [48,69,70]. The accumulation of D-2 hydroxyglutarate stimulates tumorigenic activity, mainly by way of cancer cell growth promotion and the inhibition of DNA demethylases [69,71,72].

Succinate dehydrogenase (or succinate:ubiquinone oxidoreductase or mitochondrial complex II) oxidizes succinate to fumarate and reduces FAD to FADH2 (Figure 2). This enzyme is known to have four subunits encoded by four separate genes (*SDHA*, *SDHB*, *SDHC* and *SDHD*) and has no cytoplasmic isoform (Figure 2) [64,73]. Subunits A and B have oxidative activity against succinate, while C and D reduce ubiquinone to ubiquinol [48,73]. In neuroblastoma pediatric patients, alterations in the *SDHA* and *SDHB* genes have been found. It was observed that a 3-year-old patient diagnosed with primary neuroblastoma carried the germline non-sense mutation in the *SDHA* gene, while a 5-year-old patient from a Spanish family was diagnosed with carrying a germline deletion in *SDHB* [5,74]. Apart from neuroblastoma, family members carrying a genetic alteration in oxidizing succinate dehydrogenase units A and B suffer from other cancers, such as pheochromocytoma, melanoma, renal cell carcinoma, gastric cancer, prostate cancer, breast cancer and endometrial cancer [5,74]. Both mutations reduce succinate dehydrogenase activity [5,74]. The accumulated succinate works such as an onco-metabolite triggering a hypoxia-like cell response [5,69,74]. Succinate can flux from mitochondria to cytoplasm via the dicarboxylate carrier [5,69,74]. Cytoplasmic succinate blocks hypoxia-inducible factor-1 propyl hydroxylases [5,69,74]. Consequently, the hypoxia-inducible factor-1, instead of being oxidized and transported to the proteasome, is transported to the cellular nucleus and activates the hypoxia response (including glycolysis, angiogenesis and extensive proliferation) (Figure 2) [5,69,74].

Fumarase (fumarate hydratase) catalyzes the conversion of fumarate to malate (Figure 2) [69]. Malate generation is an entry reaction for further steps in the tricarboxylic acid cycle and also for the malate–aspartate shuttle (Figure 2). Therefore, the less active fumarase mutant affects two biochemical energy production pathways [38,47,75,76,77,78,79]. A mutation in the *FH* gene is known to promote renal cell tumorigenesis and also neuro-encephalopathy and early death in childhood (*FH* deficiency syndrome) [80,81,82,83]. In clinical practice, an autosomal recessive mutation in the *FH* gene (*FH* deficiency syndrome) causes fumaric aciduria, seizures, hypotonia and developmental delay, while blood test results may indicate leukopenia and neutropenia [80,81,82,83]. Contrary to deficiency syndrome, smooth muscle tumors arise from heterozygous germ line *FH* mutations [80,81,82,83,84]. The *FH* mutation reduces mitochondrial fumarase activity, while cytoplasmic fumarase activity remains undetectable [85]. A lower fumarase activity leads to fumarate accumulation, which, just as succinate accumulation, leads to hypoxia-induced factor 1 stabilization and prioritizes glycolysis over the mitochondrial aerobic metabolism (Figure 2) [69]. Hypoxia-like conditions and metabolic shifts into glycolysis have the same outcomes as described in the *SDH* gene mutation (Figure 2) [5,69,74]. Moreover, the accumulation of fumarate leads to its chemical binding with glutathione, forming oncometabolites [84,86].

## 6. Conclusions

In this review, we discussed different molecular tools by which neuroblastoma controls energy metabolism in the microenvironment. We argued that NB derived exosomes support the transformation of healthy cells into malignant cells. We opened this review with the role of neuroblastoma-derived exosomes and closed it with known genetic mutations affecting the tricarboxylic acid cycle leading to a metabolic shift in cancer cells. A growing body of evidence has emerged indicating that energy metabolism determines the overall neuroblastoma progression.

## Figures and Tables

**Table 1:** Metabolic enzymes identified in neuroblastoma cell-derived exosomes.

Enzyme	Metabolic Pathway	References
Fatty acid synthase	Lipogenesis	[6,7,39]
Glucose-6-phosphate isomerase	Glycolysis	[6,7,35,39]
Fructose-bisphosphate aldolase	Glycolysis	[6,7,35,39]
Lactate dehydrogenase	Glycolysis	[6,7,35,39]
Citrate synthase	Tricarboxylic acid cycle	[6]
Glyceraldehyde-3-phosphate dehydrogenase	Glycolysis	[6,7,35,39]
NADH-cytochrome reductase	Electron transport chain	[6,7]
α- and β-enolase	β-oxidation	[6,35]
Aconitase	Tricarboxylic acid cycle	[6,39]
Catalase	β-oxidation	[6]
Aspartate aminotransferase	Glutaminolysis	[6,39]
Glutamate dehydrogenase	Glutaminolysis	[39]
ATP synthase	Electron transport chain	[6]
Succinate dehydrogenase	Tricarboxylic acid cycle	[6]
NADH dehydrogenase	Electron transport chain	[6]
Pyruvate dehydrogenase	Metabolic link between glycolysis/tricarboxylic acid cycle/β-oxidation	[6]
Cytochrome c and c-b1	Electron transport chain	[6,35]
ATP-dependent 6-phosphofructokinase	Glycolysis	[6]
Malate dehydrogenase (mitochondrial and cytoplasmic)	Tricarboxylic acid cycle	[6,35,39]
Enoyl-CoA hydratase	β-oxidation	[6]
Electron transfer flavoprotein	β-oxidation	[6]
Long chain-3-hydroxyacyl-CoA dehydrogenase	β-oxidation	[6]
3-ketoacetyl-CoA thiolase	β-oxidation	[6]
Phosphoglycerate kinase 1	Glycolysis	[35]
Pyruvate kinase	Glycolysis	[6,7,35,39]

## Data Availability

Not applicable.

## References

[1] Maris J.M., Hogarty M.D., Bagatell R., Cohn S.L. (2007). Neuroblastoma. Lancet.

[2] Matthay K.K., Maris J.M., Schleiermacher G., Nakagawara A., Mackall C.L., Diller L., Weiss W.A. (2016). Neuroblastoma. Nat. Rev. Dis. Primers.

[3] Pastor E.R., Mousa S.A. (2019). Current management of neuroblastoma and future direction. Crit. Rev. Oncol. Hematol..

[4] Mossé Y.P., Deyell R.J., Berthold F., Nagakawara A., Ambros P.F., Monclair T., Cohn S.L., Pearson A.D., London W.B., Matthay K.K. (2014). Neuroblastoma in older children, adolescents and young adults: A report from the International Neuroblastoma Risk Group project. Pediatr. Blood Cancer.

[5] Cascón A., Landa I., López-Jiménez E., Díez-Hernández A., Buchta M., Montero-Conde C., Leskelä S., Leandro-García L.J., Letón R., Rodríguez-Antona C. (2008). Molecular characterisation of a common SDHB deletion in paraganglioma patients. J. Med. Genet..

[6] Colletti M., Petretto A., Galardi A., Di Paolo V., Tomao L., Lavarello C., Inglese E., Bruschi M., Lopez A.A., Pascucci L. (2017). Proteomic Analysis of Neuroblastoma-Derived Exosomes: New Insights into a Metastatic Signature. Proteomics.

[7] Marimpietri D., Petretto A., Raffaghello L., Pezzolo A., Gagliani C., Tacchetti C., Mauri P., Melioli G., Pistoia V. (2013). Proteome Profiling of Neuroblastoma-Derived Exosomes Reveal the Expression of Proteins Potentially Involved in Tumor Progression. PLoS ONE.

[8] Challagundla K.B., Wise P.M., Neviani P., Chava H., Murtadha M., Xu T., Kennedy R., Ivan C., Zhang X., Vannini I. (2015). Exosome-mediated transfer of microRNAs within the tumor microenvironment and neuroblastoma resistance to chemotherapy. J. Natl. Cancer Inst..

[9] Degli Esposti C., Iadarola B., Maestri S., Beltrami C., Lavezzari D., Morini M., De Marco P., Erminio G., Garaventa A., Zara F. (2021). Exosomes from Plasma of Neuroblastoma Patients Contain Doublestranded DNA Reflecting the Mutational Status of Parental Tumor Cells. Int. J. Mol. Sci..

[10] Galardi A., Colletti M., Di Paolo V., Vitullo P., Antonetti L., Russo I., Di Giannatale A. (2019). Exosomal MiRNAs in pediatric cancers. Int. J. Mol. Sci..

[11] Colletti M., Tomao L., Galardi A., Paolini A., Di Paolo V., De Stefanis C., Mascio P., Nazio F., Petrini S., Castellano A. (2020). Neuroblastoma-secreted exosomes carrying miR-375 promote osteogenic differentiation of bone-marrow mesenchymal stromal cells. J. Extracell. Vesicles.

[12] Sullivan R., Maresh G., Zhang X., Salomon C., Hooper J., Margolin D., Li L. (2017). The emerging roles of extracellular vesicles as communication vehicles within the tumor microenvironment and beyond. Front. Endocrinol..

[13] Ciregia F., Urbani A., Palmisano G. (2017). Extracellular vesicles in brain tumors and neurodegenerative diseases. Front. Mol. Neurosci..

[14] Gebara N., Rossi A., Skovronova R., Aziz J.M., Asthana A., Bussolati B. (2020). Extracellular Vesicles, Apoptotic Bodies and Mitochondria: Stem Cell Bioproducts for Organ Regeneration. Cur. Transplant. Rep..

[15] Pavlyukov M.S., Yu H., Bastola S., Minata M., Shender V.O., Lee Y., Zhang S., Wang J., Komarova S., Yamaguchi S. (2018). Apoptotic Cell-Derived Extracellular Vesicles Promote Malignancy of Glioblastoma via Intercellular Transfer of Splicing Factors. Cancer Cell.

[16] Huang Q., Li F., Liu X., Li W., Shi W., Liu F.F., O’Sullivan B., He Z., Peng Y., Tan A.C. (2011). Caspase 3-mediated stimulation of tumor cell repopulation during cancer radiotherapy. Nat. Med..

[17] Shipley M.M., Mangold C.A., Kuny C.V., Szpara M.L. (2017). Differentiated human SHSY5Y cells provide a reductionist model of herpes simplex virus 1 neurotropism. J. Virol..

[18] Ma D., Collins J., Hudlicky T., Pandey S. (2012). Enhancement of apoptotic and autophagic induction by a novel synthetic C-1 analogue of 7-deoxypancratistatin in human breast adenocarcinoma and neuroblastoma cells with tamoxifen. J. Vis. Exp..

[19] Meehan B., Rak J., Di Vizio D. (2016). Oncosomes-large and small: What are they, where they came from?. J. Extracell. Vesicles.

[20] Al-Nedawi K., Meehan B., Micallef J., Lhotak V., May L., Guha A., Rak J. (2008). Intercellular transfer of the oncogenic receptor EGFRvIII by microvesicles derived from tumour cells. Nat. Cell Biol..

[21] Minciacchi V.R., Spinelli C., Reis-Sobreiro M., Cavallini L., You S., Zandian M., Li X., Mishra R., Chiarugi P., Adam R.M. (2017). MYC Mediates Large Oncosome-Induced Fibroblast Reprogramming in Prostate Cancer. Cancer Res..

[22] Haug B.H., Hald Ø., Utnes P., Roth S.A., Løkke C., Flægstad T., Einvik C. (2015). Exosome-like Extracellular Vesicles from MYCN-amplified Neuroblastoma Cells Contain Oncogenic miRNAs. Anticancer Res..

[23] Pollet H., Conrard L., Cloos A.S., Tyteca D. (2018). Plasma membrane lipid domains as platforms for vesicle biogenesis and shedding?. Biomolecules.

[24] Szatanek R., Baran J., Siedlar M., Baj-Krzyworzeka M. (2015). Isolation of extracellular vesicles: Determining the correct approach (review). Int. J. Mol. Med..

[25] Gupta A., Pulliam L. (2014). Exosomes as mediators of neuroinflammation. J. Neuroinflamm..

[26] Chen J., Fei X., Wang J., Cai Z. (2020). Tumor-derived extracellular vesicles: Regulators of tumor microenvironment and the enlightenment in tumor therapy. Pharmacol. Res..

[27] Yaghoubi S., Najminejad H., Dabaghian M., Karimi M.H., Abdollahpour-Alitappeh M., Rad F., Mahi-Birjand M., Mohammadi S., Mohseni F., Sobhani Lari M. (2020). How hypoxia regulate exosomes in ischemic diseases and cancer microenvironment?. IUBMB Life.

[28] Gu S., Song X., Xie R., Ouyang C., Xie L., Li Q., Su T., Xu M., Xu T., Huang D. (2020). Berberine inhibits cancer cells growth by suppressing fatty acid synthesis and biogenesis of extracellular vesicles. Life Sci..

[29] Qu J.L., Qu X.J., Zhao M.F., Teng Y.E., Zhang Y., Hou K.Z., Jiang Y.H., Yang X.H., Liu Y.P. (2009). Gastric cancer exosomes promote tumour cell proliferation through PI3K/Akt and MAPK/ERK activation. Dig. Liver Dis..

[30] Ristorcelli E., Beraud E., Mathieu S., Lombardo D., Verine A. (2009). Essential role of Notch signaling in apoptosis of human pancreatic tumoral cells mediated by exosomal nanoparticles. Int. J. Cancer.

[31] Trajkovic K., Hsu C., Chiantia S., Rajendran L., Wenzel D., Wieland F., Schwille P., Brügger B., Simons M. (2008). Ceramide triggers budding of exosome vesicles into multivesicular endosomes. Science.

[32] Falcone G., Felsani A., D’Agnano I. (2015). Signaling by exosomal microRNAs in cancer. J. Exp. Clin. Cancer Res..

[33] Zhang X., Yuan X., Shi H., Wu L., Qian H., Xu W. (2015). Exosomes in cancer: Small particle, big player. J. Hematol. Oncol..

[34] Kumar A., Deep G. (2020). Hypoxia in tumor microenvironment regulates exosome biogenesis: Molecular mechanisms and translational opportunities. Cancer Lett..

[35] Nakata R., Shimada H., Fernandez G.E., Fanter R., Fabbri M., Malvar J., Zimmermann P., DeClerck Y.A. (2017). Contribution of neuroblastoma-derived exosomes to the production of pro-tumorigenic signals by bone marrow mesenchymal stromal cells. J. Extracell. Vesicles.

[36] Ma J., Xu M., Yin M., Hong J., Chen H., Gao Y., Xie C., Shen N., Gu S., Mo X. (2019). Exosomal hsa-miR199a-3p Promotes Proliferation and Migration in Neuroblastoma. Front. Oncol..

[37] Aminzadeh S., Vidali S., Sperl W., Kofler B., Feichtinger R.G. (2015). Energy metabolism in neuroblastoma and Wilms tumor. Transl. Pediatr..

[38] Szutowicz A., Bielarczyk H., Ronowska A., Gul-Hinc S., Klimaszewska-Łata J., Dyś A., Zyśk M., Pawełczyk T. (2014). Intracellular redistribution of acetyl-CoA, the pivotal point in differential susceptibility of cholinergic neurons and glial cells to neurodegenerative signals. Biochem. Soc. Trans..

[39] Fonseka P., Liem M., Ozcitti C., Adda C.G., Ang C.S., Mathivanan S. (2019). Exosomes from N-Myc amplified neuroblastoma cells induce migration and confer chemoresistance to non-N-Myc amplified cells: Implications of intra-tumour heterogeneity. J. Extracell. Vesicles.

[40] Keerthikumar S., Gangoda L., Liem M., Fonseka P., Atukorala I., Ozcitti C., Mechler A., Adda C.G., Ang C.S., Mathivanan S. (2015). Proteogenomic analysis reveals exosomes are more oncogenic than ectosomes. Oncotarget.

[41] Nieman K.M., Romero I.L., Van Houten B., Lengyel E. (2013). Adipose tissue and adipocytes support tumorigenesis and metastasis. Biochim. Biophys. Acta.

[42] Sagar G., Sah R.P., Javeed N., Dutta S.K., Smyrk T.C., Lau J.S., Giorgadze N., Tchkonia T., Kirkland J.L., Chari S.T. (2016). Pathogenesis of pancreatic cancer exosome-induced lipolysis in adipose tissue. Gut.

[43] Wu Q., Li J., Li Z., Sun S., Zhu S., Wang L., Wu J., Yuan J., Zhang Y., Wang C. (2019). Exosomes from the tumour-adipocyte interplay stimulate beige/brown differentiation and reprogram metabolism in stromal adipocytes to promote tumour progression. J. Exp. Clin. Cancer Res..

[44] Carracedo A., Cantley L.C., Pandolfi P.P. (2013). Cancer metabolism: Fatty acid oxidation in the limelight. Nat. Rev. Cancer.

[45] Lazar I., Clement E., Attane C., Muller C., Nieto L. (2018). Thematic review series: Exosomes and microvesicles: Lipids as key components of their biogenesis and functions: A new role for extracellular vesicles: How small vesicles can feed tumors’ big appetite. J. Lipid Res..

[46] Yoshida G.J. (2020). Beyond the Warburg Effect: N-Myc Contributes to Metabolic Reprogramming in Cancer Cells. Front. Oncol..

[47] Szutowicz A., Bielarczyk H., Jankowska-Kulawy A., Pawełczyk T., Ronowska A. (2013). Acetyl-CoA the key factor for survival or death of cholinergic neurons in course of neurodegenerative diseases. Neurochem. Res..

[48] Anderson N.M., Mucka P., Kern J.G., Feng H. (2018). The emerging role and targetability of the TCA cycle in cancer metabolism. Protein Cell.

[49] Choi S.Y.C., Collins C.C., Gout P.W., Wang Y. (2013). Cancer-generated lactic acid: A regulatory, immunosuppressive metabolite?. J. Pathol..

[50] Kim S.Y. (2018). Cancer energy metabolism: Shutting power off cancer factory. Biomol. Ther..

[51] Shu S., Yang Y., Allen C.L., Maguire O., Minderman H., Sen A., Ciesielski M.J., Collins K.A., Bush P.J., Singh P. (2019). Publisher Correction: Metabolic reprogramming of stromal fibroblasts by melanoma exosome microRNA favours a pre-metastatic microenvironment. Sci. Rep..

[52] Purvis I.J., Velpula K.K., Guda M.R., Nguyen D., Tsung A.J., Asuthkar S. (2020). B7-H3 in Medulloblastoma-Derived Exosomes; A Novel Tumorigenic Role. Int. J. Mol. Sci..

[53] Yan W., Wu X., Zhou W., Fong M.Y., Cao M., Liu J., Liu X., Chen C.H., Fadare O., Pizzo D.P. (2018). Cancer-cell-secreted exosomal miR-105 promotes tumour growth through the MYC-dependent metabolic reprogramming of stromal cells. Nat. Cell Biol..

[54] Göran Ronquist K. (2019). Extracellular vesicles and energy metabolism. Clin. Chim. Acta.

[55] Oliynyk G., Ruiz-Pérez M.V., Sainero-Alcolado L., Dzieran J., Zirath H., Gallart-Ayala H., Wheelock C.E., Johansson H.J., Nilsson R., Lehtiö J. (2019). MYCN-enhanced Oxidative and Glycolytic Metabolism Reveals Vulnerabilities for Targeting Neuroblastoma. iScience.

[56] Diehl K., Dinges L.A., Helm O., Ammar N., Plundrich D., Arlt A., Röcken C., Sebens S., Schäfer H. (2018). Nuclear factor E2-related factor-2 has a differential impact on MCT1 and MCT4 lactate carrier expression in colonic epithelial cells: A condition favoring metabolic symbiosis between colorectal cancer and stromal cells. Oncogene.

[57] Edsjö A., Lavenius E., Nilsson H., Hoehner J.C., Simonsson P., Culp L.A., Martinsson T., Larsson C., Påhlman S. (2003). Expression of trkB in human neuroblastoma in relation to MYCN expression and retinoic acid treatment. Lab. Investig..

[58] Valter K., Zhivotovsky B., Gogvadze V. (2018). Cell death-based treatment of neuroblastoma review-Article. Cell Death Dis..

[59] Ren P., Yue M., Xiao D., Xiu R., Gan L., Liu H., Qing G. (2015). ATF4 and N-Myc coordinate glutamine metabolism in MYCN-amplified neuroblastoma cells through ASCT2 activation. J. Pathol..

[60] Wang T., Liu L., Chen X., Shen Y., Lian G., Shah N., Davidoff A.M., Yang J., Wang R. (2018). MYCN drives glutaminolysis in neuroblastoma and confers sensitivity to an ROS augmenting agent. Cell Death Dis..

[61] Carroll P.A., Diolaiti D., McFerrin L., Gu H., Djukovic D., Du J., Cheng P.F., Anderson S., Ulrich M., Hurley J.B. (2015). Deregulated Myc requires MondoA/Mlx for metabolic reprogramming and tumorigenesis. Cancer Cell.

[62] Jin L., Alesi G.N., Kang S. (2016). Glutaminolysis as a target for cancer therapy. Oncogene.

[63] Ciccarone F., Di Leo L., Lazzarino G., Maulucci G., Di Giacinto F., Tavazzi B., Ciriolo M.R. (2020). Aconitase 2 inhibits the proliferation of MCF-7 cells promoting mitochondrial oxidative metabolism and ROS/FoxO1-mediated autophagic response. Br. J. Cancer.

[64] Desideri E., Vegliante R., Ciriolo M.R. (2015). Mitochondrial dysfunctions in cancer: Genetic defects and oncogenic signaling impinging on TCA cycle activity. Cancer Lett..

[65] Wang W., Deng Z., Hatcher H., Miller L.D., Di X., Tesfay L., Sui G., D’Agostino R.B., Torti F.M., Torti S.V. (2014). IRP2 regulates breast tumor growth. Cancer Res..

[66] Singh K.K., Desouki M.M., Franklin R.B., Costello L.C. (2006). Mitochondrial aconitase and citrate metabolism in malignant and nonmalignant human prostate tissues. Mol. Cancer.

[67] Tsui K.H., Feng T.H., Lin Y.F., Chang P.L., Juang H.H. (2011). P53 downregulates the gene expression of mitochondrial aconitase in human prostate carcinoma cells. Prostate.

[68] Yan H., Parsons D.W., Jin G., McLendon R., Rasheed B.A., Yuan W., Kos I., Batinic-Haberle I., Jones S., Riggins G.J. (2009). IDH1 and IDH2 mutations in gliomas. N. Engl. J. Med..

[69] Sajnani K., Islam F., Smith R.A., Gopalan V., Lam A.K.Y. (2017). Genetic alterations in Krebs cycle and its impact on cancer pathogenesis. Biochimie.

[70] Han S., Liu Y., Cai S.J., Qian M., Ding J., Larion M., Gilbert M.R., Yang C. (2020). IDH mutation in glioma: Molecular mechanisms and potential therapeutic targets. Br. J. Cancer.

[71] Chandrasekharan B., Anitha M., Blatt R., Shahnavaz N., Kooby D., Staley C., Mwangi S., Jones D.P., Sitaraman S.V., Srinivasan S. (2011). Colonic motor dysfunction in human diabetes is associated with enteric neuronal loss and increased oxidative stress. Neurogastroenterol. Motil..

[72] Figueroa M.E., Abdel-Wahab O., Lu C., Ward P.S., Patel J., Shih A., Li Y., Bhagwat N., Vasanthakumar A., Fernandez H.F. (2010). Leukemic IDH1 and IDH2 Mutations Result in a Hypermethylation Phenotype, Disrupt TET2 Function, and Impair Hematopoietic Differentiation. Cancer Cell.

[73] Bardella C., Pollard P.J., Tomlinson I. (2011). SDH mutations in cancer. Biochim. Biophys. Acta Bioenerg..

[74] Gault M.D., Mandelker D., Delair D., Stewart C.R., Kemel Y., Sheehan M.R., Siegel B., Kennedy J., Marcell V., Arnold A. (2018). Germline SDHA mutations in children and adults with cancer. Cold Spring Harb. Mol. Case Stud..

[75] Zyśk M., Sakowicz-Burkiewicz M., Pikul P., Kowalski R., Michno A., Pawełczyk T. (2020). The impact of acetyl-CoA and aspartate shortages on the N-acetylaspartate level in different models of cholinergic neurons. Antioxidants.

[76] Arun P., Moffett J.R., Namboodiri A.M. (2009). Evidence for mitochondrial and cytoplasmic N-acetylaspartate synthesis in SH-SY5Y neuroblastoma cells. Neurochem. Int..

[77] Bergmeyer H.U., Rozalskis G. (1975). The Km of malate dehydrogenase from pig heart with oxaloacetate as substrate. Z Klein Chem. Klein Biochem..

[78] Bubber P., Ke Z.J., Gibson G.E. (2004). Tricarboxylic acid cycle enzymes following thiamine deficiency. Neurochem. Int..

[79] Jalil M.A., Begum L., Contreras L., Pardo B., Iijima M., Li M.X., Ramos M., Marmol P., Horiuchi M., Shimotsu K. (2005). Reduced N-acetylaspartate levels in mice lacking aralar, a brain- and muscle-type mitochondrial aspartate-glutamate carrier. J. Biol. Chem..

[80] Alam N.A., Rowan A.J., Wortham N.C., Pollard P.J., Mitchell M., Tyrer J.P., Barclay E., Calonje E., Manek S., Adams S.J. (2003). Genetic and functional analyses of FH mutations in multiple cutaneous and uterine leiomyomatosis, hereditary leiomyomatosis and renal cancer, and fumarate hydratase deficiency. Hum. Mol. Genet..

[81] Prasad C., Napier M.P., Rupar C.A. (2017). Fumarase deficiency: A rare disorder on the crossroads of clinical and metabolic genetics, neurology and cancer. Clin. Dysmorphol..

[82] Miettinen M., Felisiak-Golabek A., Wasag B., Chmara M., Wang Z., Butzow R., Lasota J. (2016). Fumarase-deficient uterine leiomyomas: An immunohistochemical, molecular genetic, and clinicopathologic study of 86 cases. Am. J. Surg. Pathol..

[83] Kamai T., Abe H., Arai K., Murakami S., Sakamoto S., Kaji Y., Yoshida K.I. (2016). Radical nephrectomy and regional lymph node dissection for locally advanced type 2 papillary renal cell carcinoma in an at-risk individual from a family with hereditary leiomyomatosis and renal cell cancer: A case report. BMC Cancer.

[84] Bardella C., Olivero M., Lorenzato A., Geuna M., Adam J., O’Flaherty L., Rustin P., Tomlinson I., Pollard P.J., Di Renzo M.F. (2012). Cells lacking the fumarase tumor suppressor are protected from apoptosis through a hypoxia-inducible factor-independent, AMPK-dependent mechanism. Mol. Cell Biol..

[85] Raimundo N., Ahtinen J., Fumić K., Barić I., Remes A.M., Renkonen R., Lapatto R., Suomalainen A. (2008). Differential metabolic consequences of fumarate hydratase and respiratory chain defects. Biochim. Biophys. Acta Mol. Basis Dis..

[86] Sullivan L., Martinez-Garcia E., Nguyen H., Mullen A., Dufour E., Sudarshan S., Licht J., Deberardinis R., Chandel N. (2013). The Proto-oncometabolite Fumarate Binds Glutathione to Amplify ROS-dependent signaling. Mol. Cell.

